# A Comparative Analysis of the Liver Retraction with Long Surgical Gauze in Three-Port Sleeve Gastrectomy and the Four-Port Nathanson Retractor Technique

**DOI:** 10.1007/s11695-024-07663-x

**Published:** 2025-01-07

**Authors:** Suleyman Caglar Ertekin, Ufuk Onsal, Emre Turgut, Huseyin Akyol, Mutlu Unver, Muhammed Taha Demirpolat, Gokhan Akbulut

**Affiliations:** 1 Altinbas University, Istanbul, Turkey; 2 Tınaztepe University Galen Hospital, İzmir, Turkey; 3 Egesehir Hospital, İzmir, Turkey; 4 University of Health Sciences, Umraniye Education and Research Hospital, Istanbul, Turkey

**Keywords:** Obesity surgery, Liver retraction, Long surgical gauze, Liver enzymes, ERAS

## Abstract

**Background:**

This study evaluated the long surgical gauze (SurG) technique as a liver retraction method in laparoscopic sleeve gastrectomy (LSG). Traditional methods involve the Nathanson retractor, associated with ischemia and necrosis complications. In addition, these techniques require an additional trocar with an incision that increases postoperative pain. Our aim, therefore, was to reduce such complications through the use of SurG and evaluate recovery and outcome implications.

**Methods:**

In this retrospective study, patients who underwent laparoscopic sleeve gastrectomy (LSG) between January and December 2023 were divided into two groups based on the liver retraction method used: NR or SurG. Demographic data, surgery times, postoperative liver enzyme levels (AST, ALT), C-reactive protein (CRP), pain scores, and analgesic use (VAS) were collected from medical records and statistically analyzed.

**Results:**

The SurG group demonstrated significantly lower postoperative pain scores and reduced analgesic use compared to the NR group (*p* < 0.001). Additionally, liver enzyme levels (AST, ALT, CRP) were lower in the SurG group, indicating less liver stress. Early mobilization was achieved more quickly in the SurG group, aligning with Enhanced Recovery After Surgery (ERAS) protocols. However, the SurG method showed some limitations during the dissection of the greater curvature due to the narrower field of view.

**Conclusions:**

The long surgical gauze method provides a viable alternative to the Nathanson retractor, offering advantages such as less postoperative pain, reduced liver stress, and quicker mobilization. Despite some technical limitations, this method can improve patient outcomes in sleeve gastrectomy.

## Introduction

Laparoscopic sleeve gastrectomy (LSG) has become one of the most commonly used surgical methods for the treatment of obesity worldwide [1–3]. One of the key challenges in laparoscopic surgery is the safe management of surrounding organs and tissues. To achieve this, surgeons typically use an assistant or a fixed retractor to carefully retract nearby organs [4].

In obesity surgery, retracting the left lobe of the liver is essential to provide a clear view of the diaphragm and upper stomach region, especially during dissection near the angle of His, a crucial area for reducing the risk of surgical complications. However, an enlarged liver can frequently obstruct this region, making the surgery more difficult [5]. One commonly used method for liver retraction is the Nathanson retractor (NR) (Cook® Medical, USA). However, this technique requires an additional incision and trocar entry and can lead to complications such as liver damage, hematoma, necrosis and epigastric pain due to prolonged pressure on the liver [6–8].

This study investigates a three-port sleeve gastrectomy technique using a long surgical gauze (SurG) (50 × 5 cm) for liver retraction instead of the NR. The primary aim of this study is to develop a technique that eliminates the need for an additional incision and trocar while reducing liver-related complications and postoperative elevations in liver enzymes. We hypothesized that this technique, which requires fewer trocars and incisions, would result in less postoperative pain, earlier mobilization, and a reduced need for analgesics on the first day compared to the standard four-port Nathanson retractor method. Additionally, we expect that the use of a SurG for retraction will decrease postoperative pain levels and accelerate the recovery process for patients.

In conclusion, this study aims to contribute to reducing retractor-related complications in obesity surgery and make the surgical process smoother with fewer trocars and incisions. This approach has the potential to enhance patient comfort and support faster postoperative recovery, offering surgeons a minimally invasive alternative.

## Materials and Methods

### Study Population and Setting

This retrospective study was conducted at Tinaztepe University Galen Hospital and Egesehir Hospital between January 2023 and December 2023; patients who underwent LSG were divided into two groups based on the method of liver retraction used. We retrospectively analyzed data collected prospectively from the patient populations in which we applied these two methods. Our clinical practice has routinely used the NR for liver retraction in LSG until May 2023. However, we hypothesized that we could get the needed retraction with a combination of a SurG and positional changes. Since June 2023, we have been using the SurG technique for retraction of the liver. Inclusion criteria for the study were patients over the age of 18 with complete data who provided informed consent. Exclusion criteria included patients with missing data, who underwent other bariatric procedures and those under 18 years of age. This study was conducted in full compliance with the ethical standards and protocols approved by the Ethics Committee of the tertiary health institution (1764.1784).

### Study Groups

Patients were divided into two groups according to the method of liver retraction used during LSG. One group underwent liver retraction using the NR, while the other group utilized SurG for liver retraction. Both groups were compared for postoperative outcomes, including pain levels, liver enzyme changes, and recovery. Both groups followed Enhanced Recovery After Surgery (ERAS) protocols.

### Surgical Technique

**Fig. 1:**
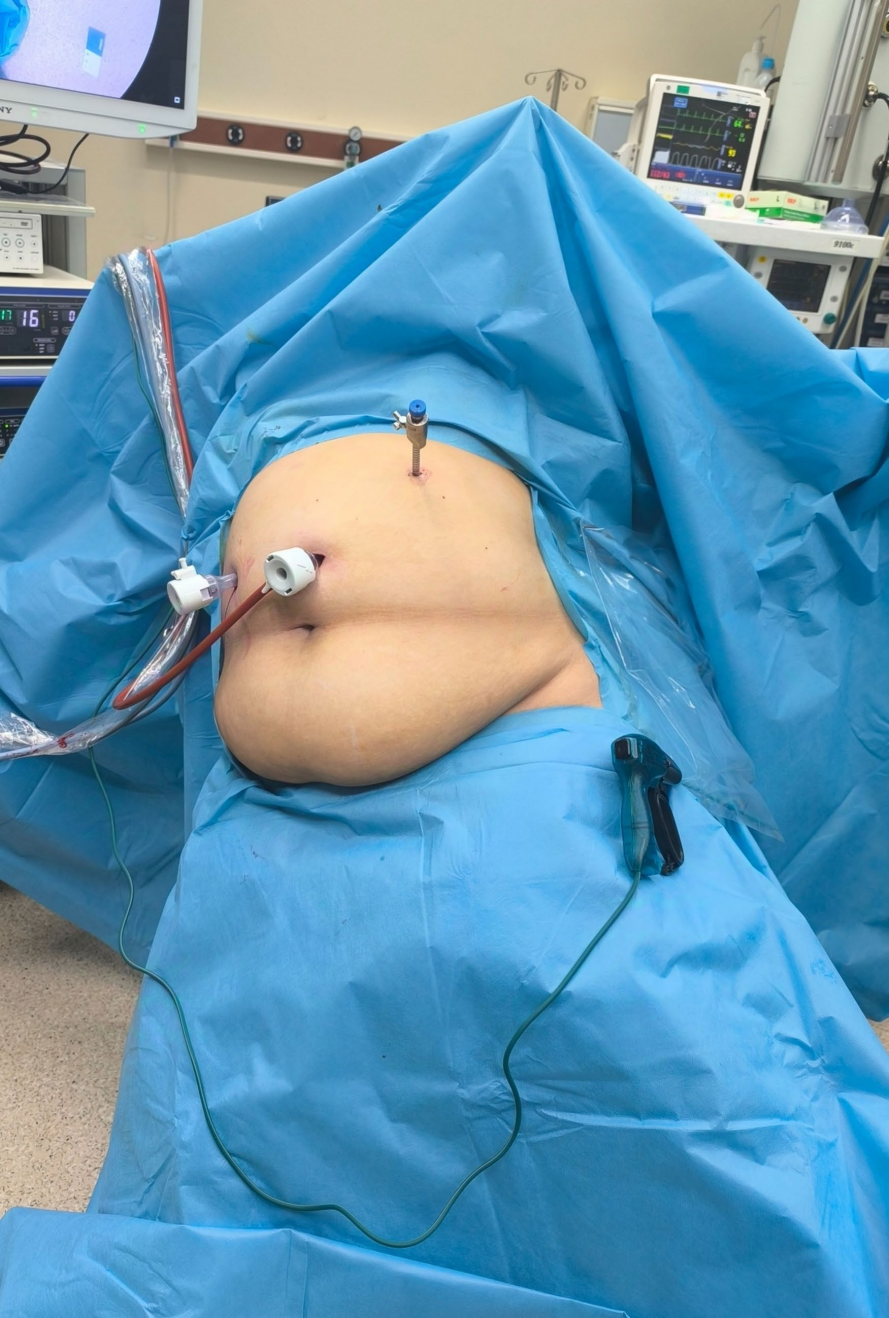
Patient positioned in reverse Trendelenburg and right-tilted lateral decubitus position to enhance liver rotation and improve surgical field visibility

**Fig. 2:**
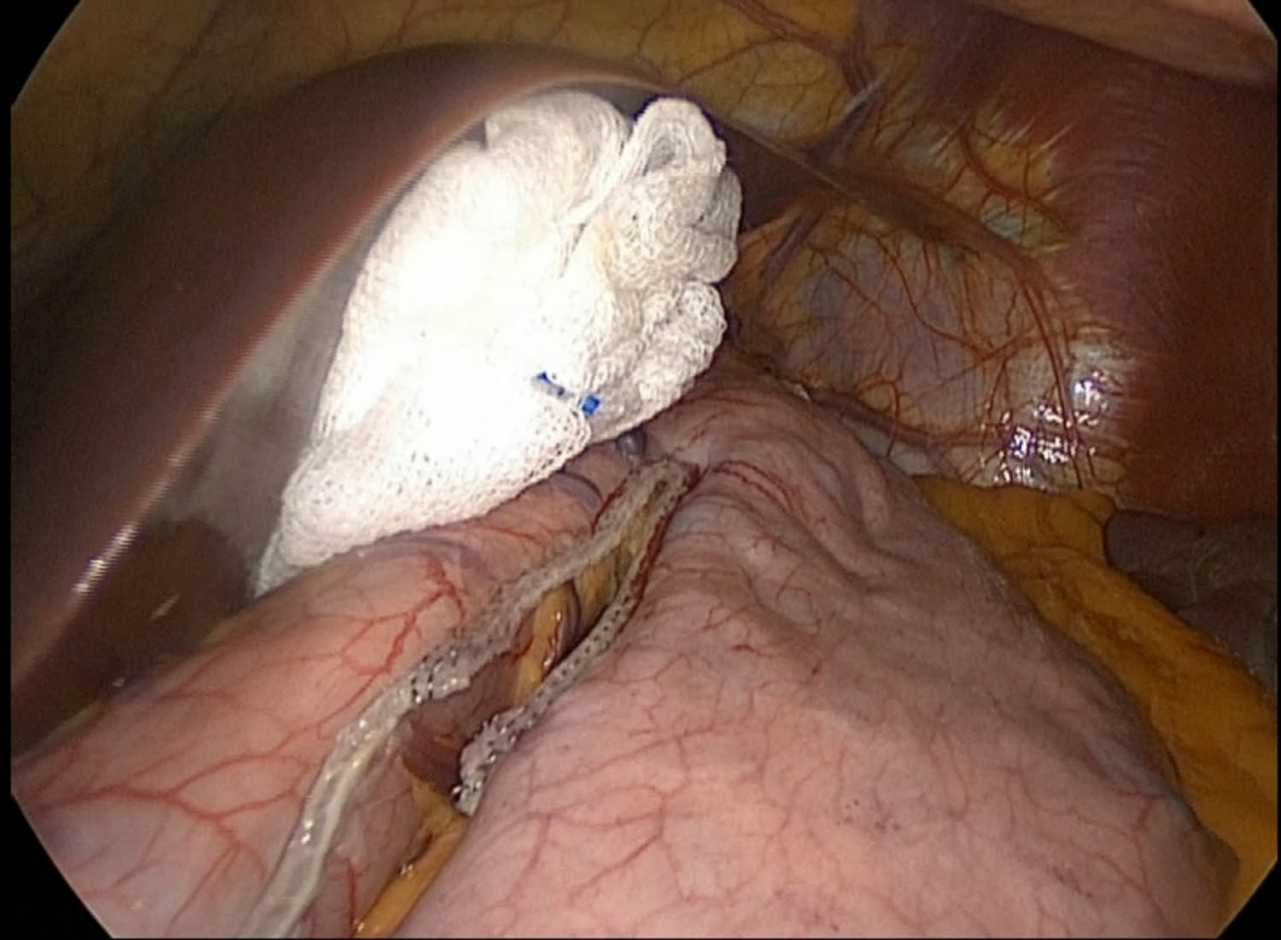
Stapling phase: surgical field and retraction with long gauze

All patients were evaluated preoperatively with gastroscopy. Sleeve gastrectomy procedures were performed using a laparoscopic approach. Initially, trocars were placed in the abdominal region. In our clinical practice, for the first trocar entry, the camera port is placed 10–12 cm below the xiphoid process and to the left of the midline. After the incision, dissection is performed with a finger down to the fascia. The fascia is held with two towel clamps, and after insufflation of the abdomen with a Veress needle, a 12-mm trocar is inserted. In the three-port technique, a 10-mm trocar was inserted above the umbilicus, a 12-mm trocar in the right upper quadrant, and a 5-mm trocar in the left upper quadrant. After obtaining an adequate view of the abdominal cavity, the patient was placed in a reverse Trendelenburg and right-tilted lateral decubitus position. This position helps rotate the liver to the right, improving the visibility of the surgical field (Fig. 1). Then, a long surgical gauze was shaped into a ball and placed between the liver and stomach, approximately 2 cm away from the hiatus, to retract the liver (Fig. 2). The gauze is inserted through a 12-mm trocar and then shaped into a ball.

In the four-port technique, an additional 5-mm trocar was inserted in the subxiphoid area, and the Nathanson retractor was used for liver retraction. In both techniques, the operative view scoring system, ranging from 1 to 5, was used to evaluate the visualization of the gastroesophageal junction (GEJ), angle of His, lesser curvature (LC), and greater curvature (GC), with 1 representing the poorest view and 5 representing optimal visibility [9].

**Fig. 3:**
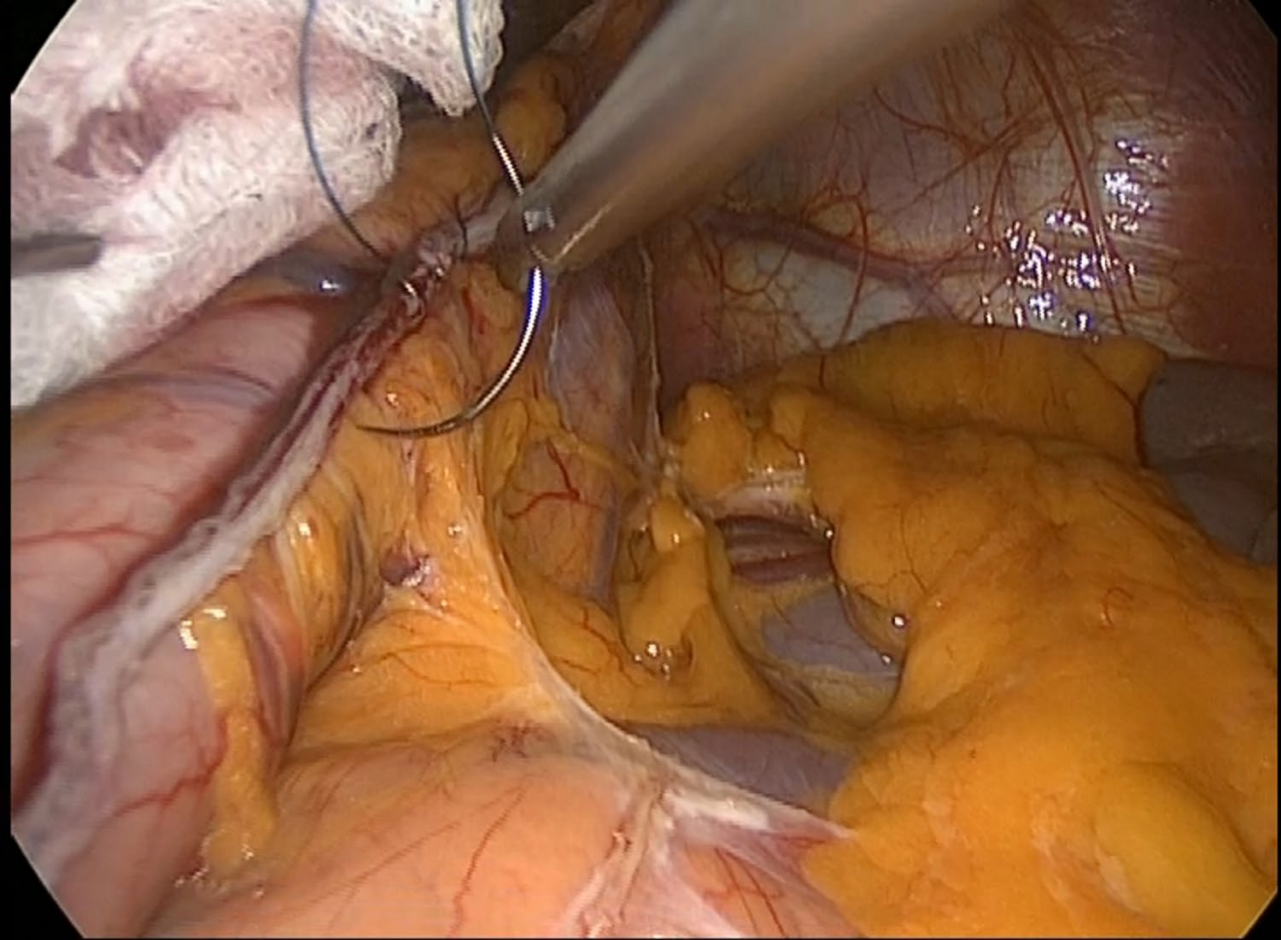
Retraction with long surgical gauze and reinforcement of the staple line with sutures

In both methods, the intra-abdominal manipulations proceeded similarly, and the stomach was carefully resected using staplers. Along the staple line, continuous sutures were placed using 3/0 absorbable barbed sutures to secure the staple line (Fig. 3). The fascia of the trocar site and camera port, where the stomach was extracted, was closed using an endoscopic fascia closure device.

### Data Collection

Data collected included demographic details, body mass ındex (BMI), hepatosteatosis (assessed by ultrasound), comorbidities (diseases), abdominal operation history, duration of surgery, staple line leaks, staple line bleeding, deep tissue infections, deep vein thrombosis, instances of liver laceration, trocar-induced hemorrhage, subxiphoid trocar site infections, and liver enzyme levels both preoperatively and at 24 and 48 h postoperatively. The enzymes measured were aspartate transaminase (AST) with normal levels of 1–35 U/L, alanine transaminase (ALT) with normal levels of 1–34 U/L, C-reactive protein (CRP) with normal levels up to 5.0 mg/L, alkaline phosphatase (ALP) with normal levels between 40 and 130 IU/L, and gamma-glutamyl transferase (GGT) with normal levels between 5 and 45 U/L. The analysis was conducted with reference to established normal ranges for each enzyme. The length of hospital stay was also assessed for both groups to determine the average hospitalization duration. Pain levels were evaluated using the Visual Analog Scale (VAS) at various time points, including preoperatively, and at 6, 12, 24, and 48 h postoperatively, as well as on the 10th postoperative day. VAS scores, surgical hematomas, bleeding, and postoperative nausea and vomiting (PONV) are routinely evaluated independently of the surgical procedure. These parameters are systematically assessed for every surgery by clinical ward and outpatient clinic nurses and are documented in patient records.

### Statistical Analysis

Baseline clinical data were analyzed using the *t*-test or Mann–Whitney *U* test for continuous variables and the Fisher’s exact test or chi-square test for categorical variables. SPSS version 22.0 (SPSS Inc., Chicago, IL, USA) was used for data analysis. Descriptive statistics (mean, standard deviation, median, frequency, percentage, minimum, maximum) were applied to evaluate the data. The one-way ANOVA test was used to compare normally distributed quantitative variables between the groups. A *p*-value of less than 0.05 was considered statistically significant.

### Outcome Measures

#### Primary Outcomes

The main endpoints of this study were to determine the reduction of liver-related complications, including ischemia, necrosis when using (SurG versus the NR). Other key outcomes were if or not SurG reduces post-operative pain as measured by the VAS at different times.

#### Secondary Outcomes

Secondary outcomes assessed the improvement of postoperative recovery, early mobilization, and lower consumption of analgesics. Cosmetic advantages regarding trocar incisions, postoperative complications such as liver lacerations, hematomas, and infections, and the quality of the operative view were also assessed. Lastly, the duration of surgery and duration of hospital stay were compared for both groups.

## Results

**Fig. 4:**
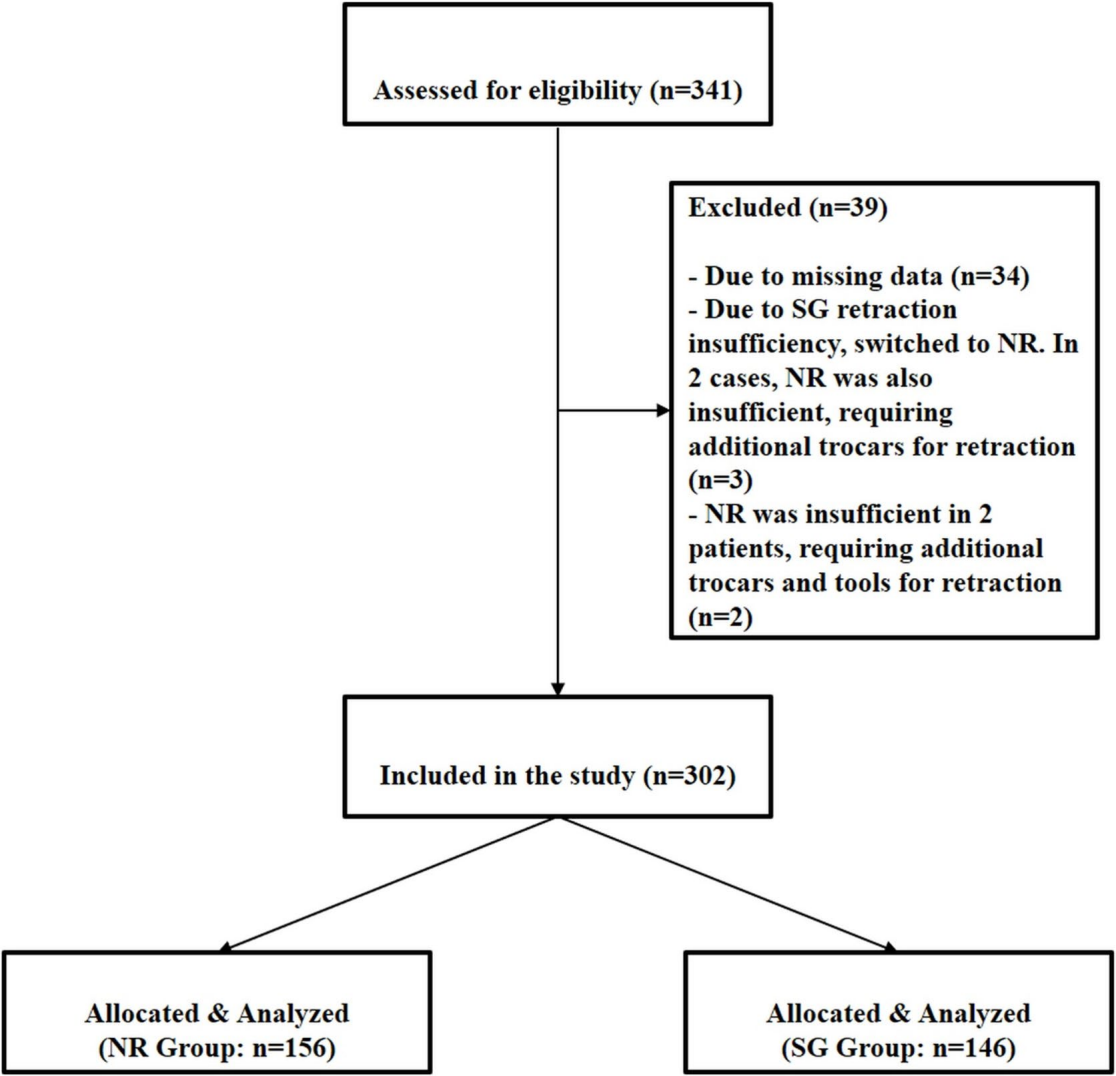
Flow chart

A total of 341 patients were analyzed, but some of these patients were excluded for various reasons. A total of 302 patients who underwent LSG between January 2023 and December 2023 were included in the study. The patient selection flowchart is shown in Fig. 4. The mean age was 35.65 ± 11.11 years in the NR group and 36.45 ± 10.39 years in the SurG group (*p* = 0.524). The sex distribution showed no significant difference (*p* = 0.645) with 75% female in the NR group and 79.5% in the SurG group. The BMI range for the operated patients was between 31 and 58.8. BMI was also comparable between the two groups (45.07 ± 5.35 kg/m^2^ for NR and 45.98 ± 4.73 kg/m^2^ for SurG; *p* = 0.121). There was no significant difference in the distribution of hepatic steatosis grades between the two groups (*p* = 0.996). The presence of diabetes (*p* = 0.278), asthma (*p* = 0.563), hyperlipidemia (*p* = 0.254), fibromyalgia (*p* = 0.342), anxiety disorders (*p* = 0.235), and HbA1c (*p* = 0.347) was statistically non-significant. However, the incidence of hypertension was significantly higher in the SurG group (46.6%) compared to the NR group (34.6%; *p* = 0.023). No significant difference was found in the operative time between the two groups, with the NR group averaging 71.24 ± 9.73 min and the SurG group averaging 69.50 ± 9.03 min (*p* = 0.110) (Table 1).

**Table 1:** Evaluation of demographic features according to the liver retraction type

Variables		Retractor type		*p* value
		Group NR (*n* = 156)	Group SurG (*n* = 146)	
Age	Mean ± SD	35.65 ± 11.11	36.45 ± 10.39	0.524
Gender	Female Male	117 (75) 39 (25)	116 (79.5) 30 (20.5)	0.645
BMI (kg/m^2^)	Mean ± SD	45.07 ± 5.35	45.98 ± 4.73	0.121
Hepatosetatosis	*n*, (%)			
• Grade 1		11 (7.1)	10 (6.8)	0.996
• Grade 2		90 (57.7)	84 (57.5)	
• Grade 3		55 (35.3)	52 (35.6)	
Diabetes	*n*, (%)	33 (21.2)	36 (24.7)	0.278
Hypertension	*n*, (%)	54 (34.6)	68 (46.6)	0.023
Asthma	*n*, (%)	11 (7.1)	10 (6.8)	0.563
Hyperlipidemia	*n*, (%)	51 (32.7)	54 (37)	0.254
Fibromyalgia	*n*, (%)	26 (16.7)	20 (13.7)	0.342
Anxiety disorders	*n*, (%)	30 (19.2)	34 (23.3)	0.235
Hemoglobin A1c (HbA1c)	Mean ± SD	6.20 ± 1.96	6.41 ± 1.88	0.347
Abdominal operation history	*n*, (%)	34 (21.8)	36 (24.7)	0.325

*BMI* body mass index

Postoperative complications, such as staple line bleeding and infections, were rare and did not significantly differ between the groups. Postoperative bleeding occurred in 0.6% of the NR group and 2.1% of the SurG group (*p* = 0.287). Deep tissue infection occurred in 1.9% of the NR group and 1.4% of the SurG group (*p* = 0.531). Liver lacerations, however, were significantly more frequent in the NR group (3.8%) compared to none in the SurG group (*p* = 0.018). Additionally, retractor-related injuries, such as epigastrica superior artery-vein injury (7.1% in NR group, 0% in SurG group, *p* = 0.001) and subxiphoid trocar site hematomas (6.4% in NR group, 0% in SurG group, *p* = 0.001), were observed exclusively in the NR group. The mean operative view score was significantly higher in the NR group (4.31 ± 0.55) compared to the SurG group (4.10 ± 0.43) (*p* < 0.001). The mean operation time was slightly longer in the NR group (71.24 ± 9.73 min) compared to the SurG group (69.50 ± 9.03 min), but this difference was not statistically significant (*p* = 0.110). PONV were similar between groups (30.8% in NR and 33.6% in SurG, *p* = 0.346). However, postoperative mobilization occurred significantly earlier in the SurG group (5.63 ± 1.21 h) compared to the NR group (6.85 ± 1.90 h; *p* < 0.001). Regarding postoperative analgesic use, the SurG group required significantly less tramadol (105.82 ± 68.10 mg vs. 172.12 ± 70.53 mg, *p* < 0.001) and pethidine hydrochloride (5.48 ± 24.29 mg vs. 26.60 ± 43.78 mg, *p* < 0.001) in the first 24 h postoperatively. The length of hospital stay did not significantly differ between the groups (2.78 ± 0.05 days in NR vs. 2.66 ± 0.05 days in SurG, *p* = 0.129). Pain levels, measured using the VAS, showed that the SurG group had significantly lower pain scores at the 6th hour (2.63 ± 1.22 vs. 4.05 ± 1.28, *p* < 0.001), 12th hour (2.08 ± 0.78 vs. 3.20 ± 0.92, *p* < 0.001), and 24th hour (1.66 ± 0.79 vs. 2.01 ± 0.96, *p* = 0.001) postoperatively. There were no significant differences at the 48th hour and 10th postoperative days (Table 2).

**Table 2:** Evaluation of postoperative complications within the 90 days, operation time, PONV, postoperative mobilization time, postoperative analgesic medication use, hospital stay, and pain scores in both groups

Variables		Retractor type		*p* value
		Group NR (*n* = 156)	Group SurG (*n* = 146)	
Complications within the 90 days	*n*, (%)			
• Staple line leaks		0	0	
• Postoperative bleeding		1 (0.6	3 (2.1)	0.287
• Deep tissue infection		3 (1.9)	2 (1.4)	0.531
• Deep vein thrombosis		0	0	
• Liver laceration		6 (3.8)	0	0.018
• Retraction related trocar artery-vein injury (A. V. Epigastrica superior)		11 (7.1)	0	0.001
• Retractor-related subxiphoidal trocar hematoma		10 (6.4)	0	0.001
Operative view scoring	Mean ± SD	4.31 ± 0.55	4.10 ± 0.43	< 0.001
Operation time (min)	Mean ± SD	71.24 ± 9.73	69.50 ± 9.03	0.110
PONV	*n* (%)	48 (30.8)	49 (33.6)	0.346
Postoperative mobilization time (h)	Mean ± SD	6.85 ± 1.90	5.63 ± 1.21	< 0.001
Postoperative analgesic medication use in the first 24 h	Mean ± SD			
• Paracetamol (mg)		31.73 ± 6.34	31.64 ± 6.43	0.906
• Dexketoprofen trometamol (mg)		177.56 ± 41.85	180.14 ± 40	0.586
• Tramadol (mg)		172.12 ± 70.53	105.82 ± 68.10	< 0.001
• Pethidine hydrohloride (mg)		26.60 ± 43.78	5.48 ± 24.29	< 0.001
Hospital stay (days)	Mean ± SD	2.78 ± 0.05	2.66 ± 0.05	0.129
Pain scores (VAS)	Mean ± SD			
• Preoperative		0.10 ± 0.37	0.10 ± 0.39	0.874
• Postoperative 6th hour		4.05 ± 1.28	2.63 ± 1.22	< 0.001
• Postoperative 12th hour		3.20 ± 0.92	2.08 ± 0.78	< 0.001
• Postoperative 24th hour		2.01 ± 0.96	1.66 ± 0.79	0.001
• Postoperative 48th hour		1.59 ± 0.77	1.58 ± 0.77	0.875
• Postoperative 10th day		0.91 ± 0.67	0.91 ± 0.67	0.922

*PONV* postoperative nausea and vomiting

Postoperatively, AST, ALT, and CRP levels were significantly lower in the SurG group compared to the NR group. At 24 h, AST levels were 30.65 ± 11.65 U/L in the NR group and 27.36 ± 14.50 U/L in the SurG group (*p* = 0.030), while at 48 h, the levels were 25.85 ± 7.85 U/L in the NR group and 22.21 ± 13.21 U/L in the SurG group (*p* = 0.004). ALT levels at 24 h were 31.31 ± 5.79 U/L in the NR group and 26.57 ± 5.30 U/L in the SurG group (*p* < 0.001), and at 48 h, they were 28.70 ± 5.10 U/L in the NR group and 25.67 ± 5.35 U/L in the SurG group (*p* < 0.001). CRP levels were also significantly lower in the SurG group at 24 h (21.24 ± 7.17 mg/L vs. 24.57 ± 6.32 mg/L, *p* < 0.001) and at 48 h (13.03 ± 6.26 mg/L vs. 21.24 ± 7.17 mg/L, *p* = 0.015). GGT and ALP levels did not differ significantly between the groups (Table 3).

**Table 3:** Evaluation of AST, ALT, GGT, ALP, and CRP measurements in pre-operative, post-operative 1 day and post-operative 2 days according to the retractor used

Variables			Retractor type		*p* value
			Group NR (*n* = 156)	Group SurG (*n* = 146)	
AST	Pre-op Post-op 1 Post-op 2	Mean ± SD	24.82 ± 7.19 30.65 ± 11.65 25.85 ± 7.85	26.90 ± 13.13 27.36 ± 14.50 22.21 ± 13.21	0.086 0.030 0.004
ALT	Pre-op Post-op 1 Post-op 2	Mean ± SD	28.53 ± 5.96 31.31 ± 5.79 28.70 ± 5.10	27.82 ± 4.71 26.57 ± 5.30 25.67 ± 5.35	0.257 < 0.001 < 0.001
GGT	Pre-op Post-op 1 Post-op 2	Mean ± SD	25.94 ± 5.27 27.96 ± 4.99 23.47 ± 5.30	26.30 ± 6.89 27.45 ± 5.81 23.69 ± 6.83	0.612 0.416 0.749
ALP	Pre-op Post-op 1 Post-op 2	Mean ± SD	68.70 ± 5.43 69.06 ± 5.21 62.35 ± 5.13	70.14 ± 6.13 69.34 ± 6.28 62.51 ± 5.92	0.030 0.670 0.803
CRP	Pre-op Post-op 1 Post-op 2	Mean ± SD	4.53 ± 3.38 24.57 ± 6.32 21.24 ± 7.17	5.03 ± 3.53 21.24 ± 7.17 13.03 ± 6.26	0.212 < 0.001 0.015

*AST* aspartate transaminase, *ALT* alanine transaminase, *ALP* alkaline phosphatase, *GGT* gamma-glutamyl transferase, *CRP* C-reactive protein, *SD* standard deviation

## Discussion

This study assessed the potential benefit of using SurG for liver retraction during LSG. In these operations, ensuring a clear view of the gastroesophageal junction and the angle of His is only possible through the safe retraction of the left lobe of the liver [10]. For this purpose, mechanical retractors such as the NR are frequently used. However, it is known that the prolonged pressure of the NR on the liver can lead to complications such as hematoma, necrosis, and segmental atrophy [11, 12]. In this study, we examined whether the results of the LSG technique using a SurG instead of NR could reduce the risk of these complications.

In our study, it has been demonstrated that the SurG offers several advantages over the traditional NR method. Liver retraction is of critical importance in bariatric surgeries and must be performed carefully to minimize complications. The SurG has been preferred due to its ability to avoid the need for an additional trocar and the lack of prolonged pressure on the liver. This technique has shown positive outcomes, such as reduced postoperative pain, decreased analgesic use, and lower postoperative liver enzyme levels. The findings indicate that the gauze method not only enhances patient comfort but also improves surgical outcomes.

Studies using the NR have reported that continuous pressure on the left lobe of the liver can lead to complications such as hematoma and necrosis [7]. In contrast, in our study, no such stress or complications were observed with the SurG. The SurG reduces the risk of complications by applying less pressure on the liver, and the absence of a need for an additional trocar not only simplifies the surgical procedure technically but also provides better cosmetic results for patients. This demonstrates that the method is a safe alternative that enhances patient comfort.

In our study, it was found that postoperative pain scores were significantly lower, especially in the first 24 h, which resulted in reduced use of analgesic medications. The use of tramadol and pethidine was significantly less in the SurG group. Similarly, previous studies have reported that the NR method increases pain scores and that additional incisions raise the pain levels during the postoperative period [9]. In a study conducted by Shinohara et al., it was reported that an alternative liver retraction method, which could replace NR, was effective in reducing postoperative pain [13]. Likewise, Sakaguchi et al. noted that new liver retraction techniques contributed to better postoperative pain control [14]. In our study, VAS pain scores were also found to be lower in the SurG group at 6, 12, and 24 h. These findings support that the gauze method is a more advantageous technique both in terms of patient comfort and the recovery process.

In the literature, it has been reported that the use of NR leads to an increase in postoperative liver enzyme levels, which is attributed to the continuous pressure applied to the liver [15]. In our study, AST, ALT, and CRP levels were found to be statistically lower in the SurG group on the 1st and 2nd days compared to the NR group. This finding indicates that the gauze method reduces the pressure on the liver and causes less liver damage. Similarly, in a study by Goel et al., it was noted that the use of NR increased liver transaminase levels and was associated with a higher risk of postoperative complications [9]. However, additional research with larger sample numbers and extended follow-up is necessary to assess the wider clinical effects of these findings, including whether the observed reduction in liver enzymes has any clinical significance.

It was also found that postoperative mobilization occurred more quickly in the SurG group, which is more compatible with ERAS protocols. Early mobilization accelerates the recovery process and reduces potential complications. Studies using the NR have reported prolonged mobilization times, leading to delayed recovery [16]. Thorell et al. emphasized that faster mobilization, a key component of ERAS protocols, improves postoperative outcomes and reduces hospital stay [17]. Similarly, Demirpolat et al. highlighted the importance of early mobilization in enhancing recovery and minimizing complications in bariatric surgery [2]. These findings suggest that the gauze method is more suitable for ERAS protocols and improves patient care in the postoperative period.

The gauze method has certain limitations. First, the patient’s position differs from the standard sleeve gastrectomy position; the right lateral decubitus position may require an adjustment period for the surgeon and the team. This position can narrow the surgical field, particularly during the dissection of the greater curvature and omentum. Additionally, the method may limit the field of view and could require some experience to manage effectively. In super-morbidly obese patients, anatomical factors could further restrict visibility, and the applicability of this technique may be limited. We do not yet have sufficient experience in this area. Nevertheless, while the operative view score in the SurG group was statistically lower than in the NR group, it still provided an adequate view for sleeve gastrectomy. The long-term results of this study included no assessment regarding both GERD and intrathoracic migration and a generally small sample that might weaken its generalizing power. Furthermore, the study was conducted by the same surgical team, which may introduce potential limitations, including selection bias. The technique may not be suitable in cases of large left lateral liver lobes, BMI > 50 kg/m2, gastric bypass, or concurrent hiatal hernia repair. Although no incident of retained gauze was encountered in this study, several such cases have been reported in the literature and must be considered a potential risk. Similarly, the number of cases required to reach proficiency with this technique remains to be established [18, 19].

## Conclusion

In sleeve gastrectomy, the long surgical gauze method used in our study demonstrated a lower risk of complications and a faster recovery process compared to the Nathanson retractor. This method is particularly advantageous in reducing postoperative pain and minimizing pressure on the liver. However, further studies are needed to evaluate its effectiveness in other surgical procedures.

## Data Availability

The datasets generated during and/or analyzed during the current study are available from the corresponding author on reasonable request.
